# Comparison of Repellency Effect of Mosquito Repellents for DEET, Citronella, and Fennel Oil

**DOI:** 10.1155/2015/361021

**Published:** 2015-10-07

**Authors:** Jong Kwang Yoon, Kang-Chang Kim, Yeondong Cho, Yong-Dae Gwon, Han Sam Cho, Yoonki Heo, Kihoon Park, Yang-Won Lee, Mijeong Kim, Yu-Kyoung Oh, Young Bong Kim

**Affiliations:** ^1^Department of Bio-Industrial Technologies, Konkuk University, Seoul 143-701, Republic of Korea; ^2^Department of Dermatology, Konkuk University Hospital, Seoul 143-729, Republic of Korea; ^3^Cosmetics Research Team, Department of Pharmaceutical and Medical Device Research, Ministry of Food and Drug Safety, Chungcheongbuk-do 363-700, Republic of Korea; ^4^Department of Pharmacy, Seoul National University, Seoul 151-742, Republic of Korea

## Abstract

To confirm that Korean Food and Drug Administration (KFDA) guidelines are applicable to test the efficacy of mosquito repellents, these guidelines were used to test the efficacy and complete protection times (CPTs) of three representative mosquito repellents: N,N-diethyl-3-methylbenzamide (DEET), citronella, and fennel oil. The repellency of citronella oil decreased over time, from 97.9% at 0 h to 71.4% at 1 h and 57.7% at 2 h, as did the repellency of fennel oil, from 88.6% at 0 h to 61.2% at 1 h and 47.4% at 2 h. In contrast, the repellency of DEET remained over 90% for 6 h. The CPT of DEET (360 min) was much longer than the CPTs of citronella (10.5 min) and fennel oil (8.4 min). These results did not differ significantly from previous findings, and hence confirm that the KFDA guidelines are applicable for testing the efficacy of mosquito repellents.

## 1. Introduction

Insect-borne diseases are a worldwide health problem, especially in tropical and subtropical climates. Mosquitoes transmit many diseases, including yellow fever, dengue hemorrhagic fever, malaria, several forms of encephalitis, and filariasis [1]. For example, malaria has been estimated to kill 3 million persons per year, including over 1 million children. Mosquito repellents may effectively protect humans from vector-borne diseases as well as other problems caused by mosquitoes.

N,N-Diethyl-m-toluamide (DEET) is a readily available and frequently used mosquito repellent. However, adverse effects of DEET have been reported, with some being severe enough to cause sensory disturbances and affect motor capacity, memory, and learning ability [2–8]. In addition, DEET is not recommended for children, because high concentrations of DEET can cause encephalopathy and other side effects [9, 10].

Botanical mosquito repellents, which cause little risk to the environment or human health, may be feasible alternatives to synthetic chemical repellents such as DEET. Thus, many people prefer to use natural repellents extracted from plants, such as citronella oil from* Cymbopogon nardus*,* p*-menthane-3,8-diol (PMD) from* Eucalyptus maculata citriodora*, and fennel oil from* Foeniculum vulgare* [11–14]. Little information is available, however, about the mosquito repellent activities of these natural and herbal-based substances. This study evaluated the repellency of commercially available natural mosquito repellents using the Korean FDA guidelines and compared their activities with that of 24% DEET.

## 2. Materials and Methods

### 2.1. Mosquitoes Used in Repellent Tests

*Aedes albopictus* (Skuse) mosquitoes were used for repellent testing. Mosquito larvae were obtained from the Division of Medical Entomology of Korea Centers for Disease Control and Prevention (KCDC). The larvae were reared at 27°C and 70% relative humidity at a dedicated facility of Konkuk University. Adult mosquitoes were fed and maintained on a 10% sucrose solution, as described previously [15].

### 2.2. Repellent Testing

Three kinds of mosquito repellents, 5% citronella (California Baby Citronella spray, California Baby, USA), 5% fennel oil (Moszero spray, Naturobiotech Co., Korea), and 24% DEET (Insectan Spray, Green Cross, Korea), were purchased. Aliquots of 1.5 mL were applied to volunteers' forearms to test repellent efficacy [16].

### 2.3. Test Cage

A test cage (40 × 50 × 40 cm) was constructed with a metal frame to make decontamination easier. All sides were covered with an observable white net to allow viewing. A fabric sleeve was added to the front side of the test cage to allow access by a human forearm.

### 2.4. Patch Tests

A patch containing repellent agent was applied to clean skin on the volunteer's forearm and allowed to remain on the skin for 48 hours. Volunteers were not permitted to remove or wet the patch during this time [17]. After 48 hours, the patch was removed by medical personnel, and initial results were determined. The patch region was marked on the forearm and results were determined 96 hours after initial patch placement.

### 2.5. Laboratory Tests of Mosquito Repellents

The repellent tests followed KFDA guidelines modified from WHOPES [21] and EPA methods [22]. Two hundred female mosquitoes (age 5–10 days), which had never received a blood meal, were placed into each test cage and starved of their sugar diet for 12 h before the test.

The arms of each volunteer were washed with unscented soap, rinsed with water, and dried for 5 min. A 1.5 mL aliquot of each repellent solution was applied evenly on the right forearm between the wrist and elbow using a pipette and allowed to dry for approximately 5 min. The untreated left arm was placed into a test cage for 3 min and the number of mosquitoes landing on that arm was counted. If fewer than 10 mosquitoes landed on that arm, the volunteer was excluded from further testing.

Repellent-treated right arms were placed into the test cage for 3 min at 1 h intervals, DEET-treated arms for 6 h, and arms treated with fennel or citronella oil for 2 h. The number of mosquitoes that landed on or bit that arm was recorded every hour.

Repellency (*R*) was calculated using the formula [23]

(1) $$\begin{array}{c} R\left(\;\%\;\right)=\left(\;C-\frac{T}{C}\;\right)\times 100\%, \end{array}$$

where *C* is the number of mosquito bites on the control arm and *T* the number of bites on the treated arm.

The complete protection time (CPT) was defined as the time the first mosquito landed on or bit a treated arm. To determine the CPT of mosquito repellents, the treated right arm of each volunteer was inserted into the test cage for 3 min. If there were no bites, that arm was reinserted at 10 min intervals until the first bite occurred.

### 2.6. Statistical Analysis

The repellency of the control and treated arms was compared using *F*-tests, with a *P* value < 0.05 considered statistically significant. SPSS was used for statistical analysis. The CPT of DEET repellent was replaced with a Kaplan-Meier survival function, since there were no bites over 6 h.

### 2.7. Ethics

The study protocol was approved by the IRB of Konkuk University Hospital (Approval number KUH 1120025). Forty-three volunteers were enrolled, all of whom provided written informed consent.

## 3. Results and Discussion

### 3.1. The Choice of Mosquito Species

To evaluate the effectiveness of repellent activity against mosquito, we performed preparatory experiments with widespread kinds of mosquitoes,* Culex pipiens, Aedes togoi, and Aedes albopictus*.* Culex pipiens*, common house mosquito, however, is not ideal for the repellency test in the laboratory setting because it fed on human only at night time due to its nocturnal characteristic. On the other hand,* Aedes togoi* showed much less biting activity compared to* Aedes albopictus* throughout the experiment setting, which is not optimal to quantify the biting rate to assess the effect of repellants. Thus,* Aedes albopictus* was chosen to evaluate the effect of repellant activities clearly in the experimental setting.

### 3.2. Patch Test for Mosquito Repellents

DEET, citronella, and fennel oil were tested on 10, 20, and 13 volunteers, respectively. Initial skin tests on volunteers' forearms were performed to assess their allergic responses to the three repellents. As determined by a dermatologist, none of the volunteers had allergic reactions at 48 h and 96 h (data not shown).

### 3.3. Repellent Effect for DEET, Citronella, and Fennel Oil

As hazards by mosquitoes have gradually increased, many kinds of mosquito repellents have been manufactured to protect humans against mosquito bites. Because mosquito repellents have played an important role in protecting humans from vector-borne diseases caused by mosquitoes, standardized guidelines are needed to evaluate the efficacy of these repellents.

In the United States, for example, repellents are tested against mosquitoes and other pests according to the guidelines of the Environmental Protection Agency (US EPA; [22]) and the American Society for Testing and Materials (ASTM; [24]). Although European guidelines have not been developed, the efficacy of these repellents has been tested according to the guidelines of the World Health Organization Pesticide Evaluation Scheme (WHOPES; [21]) and the US EPA, which are considered the international standard testing guidelines.

In Korea, the standardized guideline to test the efficacy of mosquito repellents has been established by modifying the existing EPA and WHOPES methods in 2012. In this study, we applied a laboratory test and the semifield test (data not shown) to the efficacy of DEET according to Yoon et al. [18] and botanical mosquito repellents such as citronella and fennel oils according to the KFDA guideline.

Table 1 shows the mean numbers of mosquitoes landing on untreated (control) and treated forearms of volunteers over 3 min. The mean number landing on the untreated forearms of 10 volunteers over 3 min was 16.00 ± 1.71. Testing of the repellency of treated forearms every hour for 6 h showed perfect repellency for 24% DEET over the first 3 hours. One (V10), two (V9 and V10), and six (V2, V3, V4, V6, V9, and V10) volunteers were bitten at 4, 5, and 6 h, respectively, making the repellency at these times 99.54 ± 0.46%, 97.89 ± 1.49%, and 90.33 ± 4.16%, respectively. These results indicated that 24% DEET had >90% repellency for 6 hours, with a complete protection time (CPT) of over 300 min. The other four volunteers treated with DEET (V1, V5, V7, and V8) were not bitten by mosquitoes for 6 h, so the average CPT for all 10 volunteers could not be calculated. Thus, CPT in this group was estimated using the Kaplan-Meier survival function, resulting in a CPT between 315.45 and 405.55 min at 95% confidence interval.

The use of botanical mosquito repellents has increased due to their lack of adverse effects on humans. Commercially available repellent products based on plant essential oils include extracts of basil, citronella, fennel, cedar, cinnamon, garlic, geranium, lavender, rosemary, thyme, pennyroyal, peppermint, pine, and verbena oils, which have shown repellent activity against different mosquito species as well as* Aedes albopictus *[1, 25–27]. This study tested the repellency and CPT of 5% citronella and fennel oil-containing products according to KFDA guidelines.

The repellency of 5% citronella oil was tested in 20 volunteers. When their untreated left forearms were exposed to 200 mosquitoes for 3 min, a mean (±SE) of 35.25 ± 2.81 mosquitoes landed.

To calculate the CPT, the treated right arm of each volunteer was placed into the test cage for 3 min at 10 min intervals until the first mosquito landed (Table 2). Seven volunteers (V3, V8, V10, V11, V12, V13, and V17) were bitten within the first 3 min, another 11 volunteers (V2, V5, V6, V7, V9, V14, V15, V16, V18, V19, and V20) during the second 3 min exposure period (13 min), and the last two (V1 and V4) during the third 3 min exposure (23 min). These results indicated that the average CPT of citronella oil for these 20 volunteers was 10.50 ± 1.20 min.

After completing the CPTs for each volunteer, repellency tests were performed at application and at 1 h and 2 h after treatment (Table 2). Repellency at 0 h, 1 h, and 2 h was 97.92 ± 0.69%, 71.42 ± 3.05%, and 57.73 ± 4.03%, respectively.

Repellency tests of fennel oil were performed on 13 volunteers. A mean (±SE) of 21.15 ± 0.36 mosquitoes landed on their untreated left forearms during exposure to 200 mosquitoes for 3 min (Table 3).

Testing of the CPT of citronella oil showed that nine volunteers (V1, V2, V3, V4, V6, V7, V10, V12, and V13) were bitten within the first 3 min, one (V5) was bitten during the second 3 min exposure period, and three (V8, V9, and V11) were bitten during the third 3 min exposure period. These results indicated that the average CPT of fennel oil for these 13 volunteers was 8.38 ± 1.12 min.

Repellency tests of fennel oil were performed at application and 1 h and 2 h later. Repellency at 0 h, 1 h, and 2 h was 88.57 ± 2.96%, 61.15 ± 3.85%, and 47.36 ± 5.78%, respectively.

Many plant essential oils contain volatile components, including alkanes, alcohols, aldehydes, terpenoids, and monoterpenoids, with some of these components showing a repellency effect in the vapor phase [28]. Due to their volatility, however, these components have a much shorter protection time against mosquitoes than DEET [1, 29]. Therefore, several controlled-release formulations have been developed to increase the duration of repellency [13, 30–32]. Therefore, Efficacy Data Sheets used to register repellent products with the EPA specify CPTs.

Fradin and Day [33] conducted the laboratory test with the method modified from EPA and WHOPES method as follows. 250 mosquitoes were placed in a test cage measuring 30 cm × 22 cm × 22 cm and volunteers' arms were inserted for 1 min every hour for a total of 4 h to test repellency. CPT was determined by inserting volunteers' arms for 1 min every 5 min for a total of 20 min until the first mosquito bite occurred. Using this method, the mean CPTs of 23.8% DEET and 5% citronella were 301.5 ± 37.6 min and 13.5 ± 7.5 min, respectively (Table 4).

In comparison, this study used a lower density of mosquitoes, with 200 mosquitoes in a cage measuring 40 cm × 50 cm × 40 cm, because the lower-density environment more accurately mimics the biting pressures during outdoor activities. The repellency and CPT of DEET were assessed for 3 min every 1 h for a total of 6 hours. In contrast, the repellency of citronella and fennel oils was tested for 2 h, because their repellency was approximately 50% at 2 h. The mean CPTs of DEET and citronella repellent were 360 min and 9.5 min, respectively, similar to previous findings [19, 33]. However, the CPT of 25% DEET repellents registered with the EPA was reported to be 480 min, which differed from our results (Table 1). Since four of our volunteers (V1, V5, V7, and V8) were not bitten by any mosquito 6 hours after DEET treatment, the average CPT would likely have been longer had the experiment been continued until each volunteer was bitten. Thus, the CPT measured in this study was consistent with that specified by the EPA.

The repellency and CPTs of DEET, citronella, and fennel oil, measured according to KFDA guidelines, were consistent with previous findings. KFDA guidelines will therefore be utilized to evaluate the efficacy of mosquito repellents.

## Figures and Tables

**Table 1:** Repellency and CPT of 24% DEET against *Aedes albopictus* in laboratory test.

	Untreated	Repellency (%) (±SE) at hours after treatment								CPT (min)
	*N*	0~3 h		4 h		5 h		6 h		
		*N*	*R* (%)	*N*	*R* (%)	*N*	*R* (%)	*N*	*R* (%)	
V1	20	0	100	0	100	0	100	0	100	Unknown
V2	10	0	100	0	100	0	100	1	90	360
V3	10	0	100	0	100	0	100	4	60	360
V4	25	0	100	0	100	0	100	1	96	360
V5	12	0	100	0	100	0	100	0	100	Unknown
V6	22	0	100	0	100	0	100	1	95.4	360
V7	11	0	100	0	100	0	100	0	100	Unknown
V8	15	0	100	0	100	0	100	0	100	Unknown
V9	13	0	100	0	100	1	92.3	2	84.6	300
V10	22	0	100	1	95.4	3	86.4	5	77.3	240

AVG	16 ± 1.71	0.0 ± 0.00	100 ± 0.0	0.1 ± 0.1	99.54 ± 0.46	0.4 ± 0.31	97.89 ± 1.49	1.4 ± 0.56	90.33 ± 4.16	301.45~401.55

The number (*N*) of mosquitoes landing on arm of each volunteer was counted per hour for 6 h. Repellency (*R*) was calculated each hour and complete protection time (CPT) was determined by calculating the number of minutes from the time of repellent application to the first mosquito landing.

**Table 2:** Repellency and CPT of 5% citronella oil against *Aedes albopictus* in laboratory test.

	Untreated	Repellency (%) (±SE) at hours after treatment						CPT (min)
	*N*	0 h		1 h		2 h		
		*N*	*R* (%)	*N*	*R* (%)	*N*	*R* (%)	
V1	50	0	100	7	86	12	76	23
V2	17	0	100	5	70.6	11	35.3	13
V3	33	3	90.9	11	66.7	13	60.6	3
V4	19	0	100	7	63.2	14	26.3	23
V5	18	0	100	13	27.7	14	22.2	13
V6	34	0	100	7	79.4	13	61.8	13
V7	32	0	100	4	87.5	14	56.3	13
V8	23	1	95.7	8	65.2	16	30.4	3
V9	27	0	100	10	63	8	70.3	13
V10	43	3	93	7	83.7	11	74.4	3
V11	38	2	94.7	13	65.8	12	68.4	3
V12	35	2	94.3	11	68.6	12	65.7	3
V13	31	2	93.5	11	64.5	15	51.6	3
V14	33	0	100	12	63.6	17	48.5	13
V15	45	0	100	11	75.6	18	60	13
V16	52	0	100	9	82.7	17	67.3	13
V17	27	1	96.3	9	66.7	15	44.4	3
V18	68	0	100	13	80.9	17	75	13
V19	38	0	100	8	78.9	7	81.5	13
V20	42	0	100	5	88.1	9	78.6	13

AVG	35.25 ± 2.81	0.70 ± 0.24	97.92 ± 0.69	9.05 ± 0.63	71.42 ± 3.05	13.25 ± 0.69	57.73 ± 4.03	10.50 ± 1.20

The number (*N*) of mosquitoes landing on arm of each volunteer was counted per hour for 2 h. Repellency (*R*) was calculated each hour and complete protection time (CPT) was determined by calculating the number of minutes from the time of repellent application to the first mosquito landing.

**Table 3:** Repellency and CPT of 5% fennel oil against *Aedes albopictus* in laboratory test.

	Untreated	Repellency (%) (±SE) at hours after treatment						CPT (min)
	*N*	0 h		1 h		2 h		
		*N*	*R* (%)	*N*	*R* (%)	*N*	*R* (%)	
V1	23	6	73.9	11	52.2	11	5.2	3
V2	21	3	85.7	10	52.4	7	66.7	3
V3	21	4	81	9	57.1	10	52.4	3
V4	20	1	95	13	35	20	0	3
V5	21	0	100	8	61.9	10	52.4	13
V6	20	2	90	10	50	18	50	3
V7	20	3	85	5	75	10	50	3
V8	20	0	100	10	50	10	50	23
V9	21	0	100	4	81	7	66.7	23
V10	22	4	90.9	8	63.6	10	54.5	3
V11	20	0	100	8	60	10	50	23
V12	22	2	66.7	4	81.8	8	63.6	3
V13	24	4	83.3	6	75	11	54.2	3

AVG	21.15 ± 0.36	2.23 ± 0.54	88.57 ± 2.96	8.15 ± 0.77	61.15 ± 3.85	10.92 ± 1.07	47.36 ± 5.78	8.38 ± 1.12

The number (*N*) of mosquitoes landing on arm of each volunteer was counted per hour for 2 h. Repellency (*R*) was calculated each hour and complete protection time (CPT) was determined by calculating the number of minutes from the time of repellent application to the first mosquito landing.

**Table 4:** Comparative CPT of DEET and citronella oil against mosquito bites.

Product name	Active ingredient	Percentage (%)	CPT (min)	Reference (year)
Insectan Spray	DEET	24	301.45~401.55	Yoon et al. (2014) [18]
			(360 ± 1.96)	
Aero Bug Off	DEET	25	480	EPA (2013) [20]
AquaPel 25% DEET				
Insect Repellent Pump	DEET	25	480	EPA (2013) [20]
Spray 27411				
/	DEET	25	360	Thavara et al. (2001) [19]
OFF! Deep Woods	DEET	23.8	301.5 (±37.6)	Fradin and Day (2002) [33]
California Baby Citronella spray	Citronella	5	9.5 (±1.43)	in this study
Buzz Away	Citronella	5	13.5 (±7.5)	Fradin and Day (2002) [33]

Complete protection time (CPT) was determined by calculating the number of minutes from the time of repellent application to the first mosquito landing.
